# Saffron extract feed improves the antioxidant status of laying hens and the inhibitory effect on cancer cells (PC3 and MCF7) Growth

**DOI:** 10.1002/vms3.910

**Published:** 2022-09-05

**Authors:** Reza Vakili, Mina Toroghian, Mahdi Elahi Torshizi

**Affiliations:** ^1^ Department of Animal Science Kashmar Branch, Islamic Azad University Kashmar Iran; ^2^ Department of Animal Science Ferdowsi University of Mashhad Mashhad Iran; ^3^ Department of Animal Science Mashhad Branch, Islamic Azad University Mashhad Iran

**Keywords:** Cancer cells, Egg qualitative traits, Layer, Saffron hydroalcoholic extract, Yolk colour

## Abstract

**Background:**

There have been some reports regarding supplementation of saffron petal extract on performance and egg quality in laying hens. However, the effect of saffron petal extract fed diet at different amounts on antioxidant status of laying hens and the impact of the resulting egg yolk on growth/inhibitory activity of cancer cells has not been fully studied.

**Objectives:**

The effect of dried saffron petal extract on the laying performance, egg qualitative traits, antioxidant status, and its inhibitory effect on cancer cells was studied.

**Methods:**

A total of 200 39‐week‐old Hy‐line W36 Leghorn laying hens were selected based on a completely randomised design in four treatments with five replications (10 hens per replication). The four treatment diets consisted of a basal diet with no supplement (control), and three diet supplement groups containing 40, 60 and 80 ppm of saffron petal extract.

**Results:**

Adding 80 ppm of saffron petal extract to layer diets increased egg production (*p* < 0.05). Malondialdehyde,1,1‐diphenyl‐2‐picrylhydrazyl value and Superoxide dismutase significantly improved by saffron petal dietary supplementation. The yolk weight and colour, Haugh unit and shell weight and thickness were also influenced (*p* < 0.05) with highest values achieved in the 60 ppm saffron extract supplemented diet. Results demonstrated a significant effect of saffron extract inclusion in the diet on the growth of Michigan Cancer Foundation‐7 and Prostate Cancer Cell in a positive dose‐dependent manner (*p* < 0.05) and the most intense inhibitory effect on cancer cells was observed with 60–80 ppm extract.

**Conclusions:**

Saffron petal extract can be used to potentially modulate the antioxidant status of laying hens and the inhibitory effect on cancer cells, best achieved with 60–80 ppm extract.

## Graphical abstract

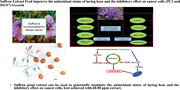

## 1 INTRODUCTION

Plants are rich in several phytochemicals or bioactive chemicals and incorporated into the animal feed for enhance productivity (Moharreri et al. (2022). Saffron has traditionally been applied as a herbal medicine. More than 150 different compounds involving carbohydrates, polypeptides, lipids, minerals and vitamins are found in saffron (Khorasany & Hosseinzadeh, 2016). Saffron contains picrocrocin, safranal, crocetin, α‐crocin, lycopene, zeaxanthin and α‐ and β‐ carotenes. As shown, it is rich in elements like Zn, Fe, Cu, Se, Mg, P, Ca and Mn, and several vitamins such as vitamin A and C, folic acid, riboflavin and niacin (Qadir et al., 2020). Saffron (*Crocus sativus*) has been considered as an additive that improves egg quality (Botsoglou et al., 2010) and oxidative stability of egg yolk (Botsoglou et al., 2005a, 2005b). Saffron petal, as an important by‐product of saffron, is annually produced in large amounts (more than 10,000 tons/year) and usually discarded as a waste product (Kafi et al., 2006). The antioxidant properties of saffron petal (Goli et al., 2012; Serrano‐Díaz et al., 2012) may be related to its phenolic compounds such as crocin and kaempfrol (Zeka et al., 2015). Different pharmacological properties of saffron petal such as antibacterial, antispasmodic, immunomodulatory, antitussive, antidepressant, antinociceptive, hepatoprotective, renoprotective, antihypertensive, antidiabetic and antioxidant activity have been reviewed (Hosseini et al., 2018). Intake some of feed additives is always considered to be important in improved performance. Bentonite prevents the effects of aflatoxin present in feed that could increase uric acid absorption from the intestine (Khanedar et al. (2013), and hydroethanolic saffron petals' extract reducing the associated oxidative damages in challenged by aflatoxin (Hosseini‐Vashan et al., 2018). Adding saffron petal powder to broiler diets decreased feed conversion ratio and increased feed intake, while bursa of Fabricius, body weight as well as ventricular fat were not influenced (Naghous et al., 2015). Incorporation of hydroalcoholic extract of saffron petals into quail diet enhanced the relative weight of lymphatic organs, body weight, and feed intake, while ventricular fat and feed conversion ratio decreased (Hosseini‐Vashan et al., 2018). In addition, dietary inclusion of adding saffron petals decreased yolk cholesterol and increased yolk colour in laying hens (Jabbari Namroudi et al., 2021).Therefore, the present study aimed to evaluate the effects of saffron hydroalcoholic extract at various concentrations on egg qualitative traits and production function, antioxidant status, as well as the impact of the egg yolk containing saffron on growth/inhibitory activity of Michigan Cancer Foundation‐7 (MCF7) and Prostate Cancer Cell (PC3).

## 2 MATERIALS AND METHOD

### 2.1 Study design

In the current study, 200 39‐week‐old Hy‐line W36 Leghorn laying hens based on a completely randomised design (CRD) were allocated into four treatments with five repetitions (10 hens per replication).This experiment was performed in the poultry house. The hens were kept in wire cages 52 cm long, 34 cm wide and 30 cm high on three floors with a maximum temperature of 20°C, a humidity of 57%, and a lighting period of 16 h. There were two nipple drinkers in each cage, and the feed was freely available to the hens.

### 2.2 Diets

The diets were adjusted based on the requirements recommended for the strain Hyline by using UFFDA software, the components of which are presented in Table 1. The present experiment was performed for 12 weeks since the beginning of laying hen production until the end of its peak.

**TABLE 1:** Basal diet composition (g/kg as fed)

Ingredients	Quantity (g/kg)
Corn	495
Soybean	280
Wheat	100
Calcium carbonate	85
Oil	8
Dicalcium phosphate	21
Sodium chloride	2.5
Sodium bicarbonate	1.5
Vitamin supplement^a^	2.5
Mineral supplement^b^	2.5
Methionine‐DL	2
Calculated and analysed chemical composition of diets (g/kg DM feed)	
Metabolisable energy (kcal/kg)	2696
Crude protein	172.4
Calcium	36.9
Available phosphorous	4.93
Sodium	1.8
Chlorine	1.8
Methionine	4.6
Methionine‐cysteine	7.44
Lysine	9.03
Threonine	6.5

^a^ In 1 kg of diet, vitamin supplement is composed of retinyl acetate: 3200,000 IU, cholecalciferol: 1320,000 IU, dl‐α‐tocopheryl acetate E: 8000 IU, Menadione: 1000 mg/kg, 3200 mg/kg calcium panthothenate, 12,000 mg/kg of niacin, 1000 mg/kg of vitamin B1, ‐2200 mg/kg of B2, 1600 mg/kg of B6, 360 mg/kg of B9, 9 mg/kg of B12, 30 mg/kg of biotin, 44,000 mg/kg of choline and 3000 mg/kg of antioxidant.

^b^ Mineral supplement contains 32000 mg of zinc, 80000 mg of manganese, 32000 mg of copper, 480 mg of iodine, 88 mg of selenium, and 16000 mg of iron per kg of basal diet.

Further, There were four experimental treatments that consisted of a basal diet + 0 (control), 40, 60 or 80 ppm of saffron petal extract.

### 2.3 Extraction from saffron petals

The first part of the study focused on the preparation of saffron petal extract. Petals, style, and stigma were shade‐dried, ground, shaken with 50% ethanol and filtered. Extraction was carried out using saffron petal and 50% aqueous ethanol solvent in a ratio of 1:10 for 2 h, followed by concentration, solvent separation, spray‐drying, sieving, and storage. The ethanolic saffron petal extract (SPE) was prepared by dissolving 100 g of the dried petal powder in 1000 ml of ethanol (50% v/v) and shaking for 2 h (GFL Orbital Shaker 3005, Burgwedel, Germany) at room temperature. Then, the extract was filtered through a Whatman No. 1 paper (Whatman Ltd., Maidstone, England). The residual solvent of the ethanolic extract was removed under reduced pressure at 38°C using a rotary evaporator (Heidolph Laborota 4000, Schwabach, Germany). The condensate extract was completely dried using a freeze‐drying system (Martin Cherist, Beta 2–8 LD plus, Osterode am Harz, Germany). The final powdered extract was then weighed to calculate the ethanolic SPE yield (w/w), which was 42%. The extract powder was stored in dark bottles at 4°C until use. In this regard, the content of kaempferol and crocin, anthocyanin, and total phenolic compounds was respectively measured according to Zeka et al. (2015) and Goli et al. (2012).

### 2.4 Total anthocyanin content

Total anthocyanin content (TAC) was evaluated by pH differential method (Giusti & Wrolstad, 2003). Hence, 0.2 g of each sample was dissolved in 10 ml of distilled water in a volume flask far from the light. Absorbance was measured at 510 nm and 700 nm.

### 2.5 Total phenolic content (TPC)

Folin–Ciocalteau method was used to measure the total phenolic content (TPC) of the extracts (Singleton et al., 1999). TPC was presented as mg gallic acid (GA) equivalents per g. For 100 μl test sample solution made from 100 mg POPx in 10 ml of methanol, 6 ml of double‐distilled water and 500 μl of Folin–Ciocalteau reagent were added.

### 2.6 Determination of total flavonol content (TFC)

The TFC content was determined according to the colorimetric assay of Spigno et al. (2007). Briefly, 0.5 ml of extracts was added into a 2.5 ml of water. At zero time, 150 μl of 5% NaNO2 was added to the mixture. After 6 min, 0.3 ml of 10% AlCl3 was added into the flask. At 5 min, 1 ml of 1 M NaOH was added to the mixture. The reaction flask was immediately diluted to volume with the addition of 550 μl of distilled water and thoroughly mixed.

### 2.7 Measurements

#### 2.7.1 Egg production performance

Diet was formulated according to guidelines and was ad libitum provided. Daily feed intake (g), egg production (% /hen/ day production) and feed conversion ratio were calculated during the experiment. In each treatment, daily feed intake was determined by subtracting the feed level remaining at the next day from the total feed for the treatment. A digital scale with the precision of 0.01 was applied to measure egg weight. The weights of feed and eggs were recorded using an electronic weighing scale with an accuracy of 10 kg × 0.5 g (Model DT580, Atrontec Electronic Tech Co., Ltd., Jiangsu, China).

#### 2.7.2 Qualitative traits of egg

In each month, four eggs were selected from each repetition and weighted to assess some qualitative traits. Then, egg shape, yolk index and colour (Vuilleumier, 1969), shell thickness (mean shell thickness in the wide and narrow end of egg, as well as its equator with the precision of ± 0.01 mm), shell weight (±0.01 g), Haugh unit and egg weight were measured (Holder & Bradford, 1979).

Shape index was obtained by using the following equation.

(1) $$SI=\left(EW/EL\right)\times 100,$$

where *SI* represents shape index, *EW* refers to egg weight and *EL* refers to its length.

#### 2.7.3 Antioxidant activities

To evaluate the antioxidant status, the activity of serum superoxide dismutase (SOD) enzyme was evaluated using Randsel SOD diagnostic kit (Randox, Crumlin, Uk) in Mad laboratory. Malondialdehyde was determined as the final product of lipid peroxidation in chicken hepatocytes using thiobarbituric acid reactant (TBARS). The measurement of free radical scavenging activity was performed according to previous research (Xie, 2014).

#### 2.7.4 Cell culture tests

The cancer cell Culture tests were carried out in the cell lab of Industrial Fungi Biotechnology Research Department, Iranian Academic Center for Education, Culture and Research (ACECR) ‐ Mashhad Branch, Khorassan Razavi, Iran according to the previously‐reported method (Rezaeian & Pourianfar, 2018). Based on the methods, MCF7 and PC3 cells were cultured in RPMI and DMEM media, respectively. Both media (as a solution containing sodium bicarbonate) were mixed with heat‐inactivated FBS (10%), filtered through a 0.22‐μm filter, and utilised for cell culture. The cells were cultured in the sterile flasks having filter cap with the growth area of 75 cm^2^ by considering the experiences acquired by cell passage. Then, they were frozen and reproduced as detailed in the previous study (Rezaeian & Pourianfar, 2018). Briefly, the freezing process was employed for the cells at exponential growth phase in a fresh freezing medium consisting of FBS and DMSO (Merck, Darmstadt, Germany) in a 9:1 ratio. Finally, the effect of egg yolk at different concentrations on the inhibition of cancer cell growth and proliferation was evaluated.

#### 2.7.5 Statistical analysis

This experiment was conducted in the form of simple CRD and CRD with several observations (qualitative traits of egg) in each replication by using the following models, respectively.

(2) $$Y_{ij}=\mu +T_{i}+e_{ij},$$

(3) $$Y_{ijk}=\mu +T_{i}+e_{ij}+\varepsilon_{ijk},$$

where *μ* demonstrates mean trait, *T_i_* indicates the effect of treatment and *ɛ_ijk_* and *e_ijk_* illustrate the effect of sampling and experimental error, respectively. [Correction added on 23 September 2022, after first online publication: Equation 3 was corrected.] The data were analysed by using GLM in SAS statistical software. Duncan test was utilised to compare the mean of various treatments when the mean difference was significant at *p* < 0.05.

Additionally, the statistical analysis of cell culture was carried out in SPSS 22 software. All of the cell tests were performed with at least three completely independent replications on separate days according to the instruction for cell cultures (Lazic et al., 2018). In each independent repetition (each 96‐well plate), three wells were assigned to each treatment (as sub‐sample). The main factor as a treatment (independent variable) included four diets, which was studied in the form of the eggs from the chickens fed with the diets. Furthermore, yolk was removed and tested as a treatment on the cell. The trait under study (dependent variable) was the level of the decrease in cell growth compared to the negative control (untreated).Two different cell lines were examined, to which each diet level was provided in five consecutive concentrations. The statistical analyses were implemented to assess the effect of diet level on cell growth reduction.

## 3 RESULTS AND DISCUSSION

Table 2 summarises the active components or secondary metabolites of saffron petal extract.

**TABLE 2:** Major bioactive constituents (secondary metabolites) of Iranian saffron petal extract

Constituent	Content
Total phenolic compounds (mg)	3.42 ± 0.11
Total flavonoids (mg/g)	2.75 ± 0.07
Kaempferol (% w/w)	12.6 ± 0.12
Crocin (% w/w)	0.6 ± 0.03
Anthocyanin (mg/L extract)	1712 ± 0.24

Treatment 1: control (basal diet); Treatment 2: basal diet + 40 ppm of saffron supplement; Treatment 3: basal diet + 60 ppm of saffron supplement; Treatment 4: basal diet + 80 ppm of saffron supplement.

The total phenolic and flavonoid contents of saffron petal in this study were 3.42 [mg of gallic acid equivalents (GAE) per gram of the extract] and 2.75 [mg of quercetin equivalents (QE) per gram of the extract], respectively. Goli et al (2012) reported total phenolic contents of 3.35 mg/g for Iranian saffron petal. Significantly greater amounts of total phenolic components (65.34 mg of GAE/g of powder extract) and flavonoids (60.64 mg CE/g of dry plant material) have been reported by Jadouali et al. (2018).The reasons for the differences between our reported values and that of other researchers are probably attributed to the genetic variation, ecological conditions and/or the differences in the extraction method.

Table 3 outlines the effects of saffron petal hydroalcoholic extract on egg production performance in layers. As shown, a significant increase was observed in egg production percentage (*p* < 0.05) in the treatment 4. Adding 80 ppm of saffron petal extract to layer diets increased egg production due its bioactive compounds, minerals and various vitamins that affects feed intake and with a positive effect on nutrient absorption efficiency improves body weight and feed conversion ratio. In addition, saffron petals had high levels of camphor, flavonoids, quercetin and minerals, which decreased the activity of free radicals and break down fats, leading to an increase in the availability of nutrients, along with the growth of poultry (Hosseini & Mollafilabi, 2017; Omidi et al., 2014). However, the treatments had no effect on feed intake, conversion ratio, and egg weight and mass (*p* > 0.05). Linear and quadratic effects were also not significant.

**TABLE 3:** Effects of saffron petal hydroalcoholic extract on egg production performance in layers

	Total period				
Treatment	Feed intake (g/days)	Conversion ratio	Egg weight (g)	Egg production (%)	Egg mass (%)
Control (basal diet)	116.245	2.283	58.657	87.2^b^	51.153
Basal diet + 40 ppm of saffron supplement	107.967	2.138	58.359	86.6^b^	50.557
Basal diet + 60 ppm of saffron supplement	108.709	2.181	59.099	84.6^b^	49.991
Basal diet + 80 ppm of saffron supplement	106.623	2.026	58.561	90.0^a^	52.719
SEM	3.566	0.091	0.462	2.2	1.299
*p* value	0.259	0.292	0.717	0.0001	0.502
Linear	0.096	0.09	0.83	0.53	0.49
Quadratic	0.398	0.95	0.79	0.21	0.22

^a,b^A statistically significant difference is observed in the means with various letters in each row (*p* < 0.05).

SEM, standard error of the mean.

The effects of saffron petal hydroalcoholic extract on the qualitative traits of the egg from layers are summarised in Table 4. Based on the results, shape index was not affected by the treatments (*p* > 0.05). In addition, the treatments influenced on egg qualitative traits from layers significantly (*p* < 0.05). The highest yolk weight was related to the treatment 3, while the traits were minimised in the first and fourth ones, respectively. Yolk colour improved in the treatment 3 and 4 significantly (*p* < 0.05). No significant differences for ‘thickness’ compared to the control. However, the treatments were not significantly different compared to the others. Further, the highest and least levels of Haugh units were respectively obtained following #1 and #4 treatments, respectively, while those of shell weight were related to the #1 and #3, respectively.

**TABLE 4:** Effects of saffron petal hydroalcoholic extract on the qualitative traits of the egg from layers

Treatment	Shape index	Yolk weight (g)	Shell weight (g)	Shell thickness (mm)	HAUGH unit	Yolk colour
Control (basal diet)	80.305	14.811^c^	5.825^a^	0.468^ab^	94.494^a^	5.7^b^
Basal diet + 40 ppm of saffron supplement	79.531	15.781^ab^	5.310^b^	0.439^b^	89.857^b^	5.9^b^
Basal diet + 60 ppm of saffron supplement	79.137	16.029^a^	5.684^a^	0.487^a^	90.853^b^	7.7^a^
Basal diet + 80 ppm of saffron supplement	79.896	15.144^bc^	5.629^ab^	0.456^ab^	96.905^a^	8^a^
SEM	0.530	0.238	0.113	0.238	1.162	1
*p* Value	0.468	0.008	0.033	0.023	0.002	0.001
Linear	0.66	0.28	0.65	0.65	0.25	0.01
Quadratic	0.36	0.01	0.04	0.32	0.01	0.76

^ab^ A statistically significant difference is observed in the means with various letters in each row (*p* < 0.05).

SEM, standard error of the mean.

Namroudi et al. (2021) reported that the use of saffron petal in diet can influence yolk colour so that its colour was significantly enhanced following the addition of 2–3% of the petal compared to the control. Botsoglou et al. (2005b) also found the yolk colour to improve significantly in the saffron group compared to other groups. The results of another experiment earlier by Botsoglou et al. (2005b) also suggested an increase in yolk colour by after the addition of saffron as a dietary supplement.

Linear effect for colour yolk and Quadratic effects for yolk weight, Haugh unit, and shell weight was significant. Saffron is rich in carotenoids (Bolhassani et al., 2014), like crocin and crocetin that are considered to be the main and crocin and crocetin as the two main natural carotenoids include were considered to be the main contributor for the colour enhancement (Bolhassani et al., 2014; Daniel, 2006; Mohajeri et al., 2010). Thus, this improvement in yolk colour reflects the transfer of crocins, lycopenes and carotenes, as the colouring constituents of saffron, from chicken diet to egg yolk (Tarantilis et al., 1995).

### 3.1 Cell culture

At First, The effect of concentration in comparison with the negative control for each treatment and each cell line were investigated. In all cases, the *F*‐test was significant (*p* < 0.01), indicating that there is at least one concentration in each treatment and in each cell line reducing the growth of cancer cells, which is significant compared to the negative control. After F‐test became significant, mean comparison tests were evaluated using Duncan's method at a significance level of 0.05 to determine the best concentration in each treatment in each cell line. It should be noted that *F*‐test and comparison of means on raw numbers (cell uptake values ​​related to treatment and cell uptake values ​​related to negative control) were performed to increase the accuracy before their conversion to percentage.

### 3.2 Effect on MCF7 cell activity

**FIGURE 1:**
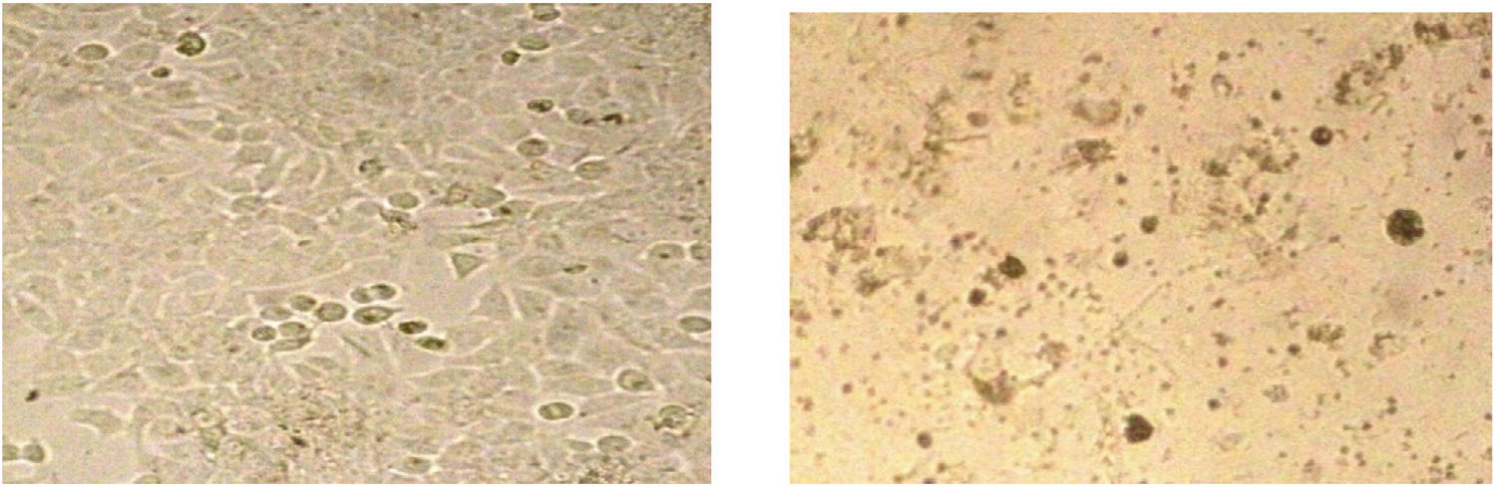
Microscopic image related to the growth reduction effects of the yolk received the treatment 4 at the concentration of 5000 μg/ml on MCF7 cancer cells (right) compared to the negative control (left), which was taken 72 h after treatment and a few min before MTT when the cells were washed with PBS buffer

Table 5 lists the mean inhibition capacity (percentage relative to the negative control) of the various concentrations of the egg yolk obtained from hen treated with different diets (T1–T4) against MCF7 cancer cell activity. As demonstrated, all treatments at various concentrations of egg yolk affected the inhibition action against MCF7 cancer cell significantly (*p* < 0.05). In Treatment T1, the inhibition was maximised at 625 and 1250 μg/ml concentrations, while the actual highest level was observed at 625 and 2500 μg/ml in T2. Additionally, the other treatments led to the high inhibition at 5000 μg/ml of egg yolk. However, there was a significant difference between the concentrations of 5000 μg/ml compared to the egg yolk obtained using T4 (Figure 1).

**TABLE 5:** Mean inhibition (percentage relative to the negative control) of the various concentrations of the egg yolk from different diets against MCF7 cancer cell (*n* = 3, *p* ≤ 0.05)

	Concentration (**μ**g/ml)				
Treatment	0 (negative control)	625	1250	2500	5000
Control (basal diet)	0^c^	12.09 ± 0.82^a^	10.43 ± 2.97^a^	8.67 ± 1.70^ab^	5.90 ± 2.93^b^
Basal diet + 40 ppm of saffron supplement	0^c^	14.85 ± 1.18^a^	10.10 ± 1.45^b^	14 ± 3.15^a^	9.02 ± 2.90^b^
Basal diet + 60 ppm of saffron supplement	0^c^	10.41 ± 0.17^ab^	8.19 ± 1.08^b^	8.56 ± 2.32^b^	12.61 ± 1.43^a^
Basal diet + 80 ppm of saffron supplement	0^c^	14.61 ± 1.67^b^	4.69 ± 0.54^c^	4.90 ± 0.42^c^	39.39 ± 3.92^a^

^a, b^ A statistically significant difference is observed in the means with various letters in each row (*p* < 0.05).

Based on the results of comparing the three diets T1, T2 and T3, the percentages of inhibition on MCF‐7 cancer cells in different concentrations were not significant. For example, in these diets, higher egg yolk concentrations may even contribute to the growth of cancer cells due to the excessive nutrients available in the egg yolk. In the study of Lee & Paik (2019), the results indicated that there are anticancer activities of egg proteins and associated peptides, which can occur via a variety of mechanisms.

In addition, a significant difference was observed between the concentration of 5000 μg/ml compared with other concentrations, especially in T4 (Figure 1), which may be related to the anticancer compounds in the egg yolk of T4 due to feeding laying hens with the highest dose of saffron petal extract, along with the highest concentration of egg yolk of 5000 μg/ml which could have a natural positive effect of cell culture suppression.

Quercetin and its analogues can inhibit the proliferation of breast cancer cells. In the present study, a dose‐dependent inhibition cancer MDA‐MB‐ and 231 MCF was observed in cell proliferation. In another study, quercetin had a proapoptotic effect on MCF‐7, which indicates the existence of a Caspase‐dependent pathway through increased protein expression of BAX (Chryssanthi et al., 2007).

### 3.3 Effect on PC3 cell activity

**FIGURE 2:**
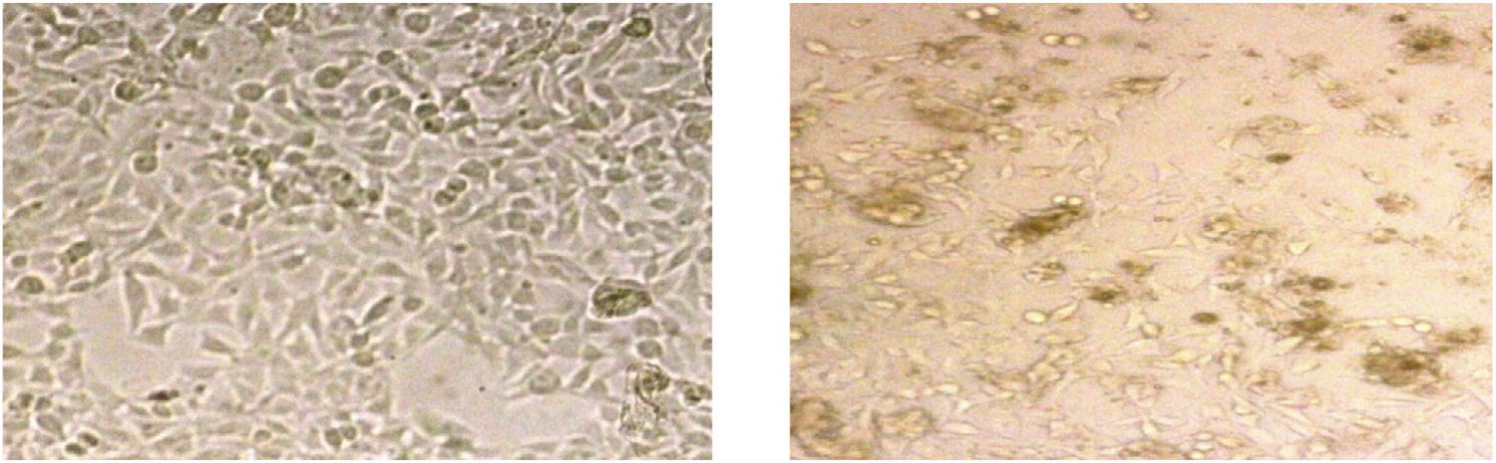
Microscopic image related to the growth reduction effects of the yolk received the treatment 4 at the concentration of 5000 μg/ml on PC3 cancer cells (right) compared to the negative control (left), which was taken 72 h after treatment and a few min before MTT when the cells were washed with PBS buffer

Table 6 represents the mean inhibition percentage relative to the negative control of the different concentrations of the egg yolk from various diets against PC3 cancer cell. The results of mean comparison indicated a higher inhibition percentage at the concentrations of 1250, 2500 and 5000 μg/ml in the treatment T1 compared to the negative control and 625 μg/ml concentration treatment. However, the three higher concentrations were not significantly different (*p* > 0.05). Following the second treatment T2, the concentrations of 1250 and 5000 μg/ml resulted in maximising the percentage inhibition. Although the difference was insignificant (*p* > 0.05). The highest and lowest inhibition percentages in the last treatment T4 was 5000 μg/ml so that an increase in concentration led to an increase in the percentage inhibition. In this treatment, a significant inhibition was observed at 5000 μg/ml, which is consistent with those obtained for MCF7 (Figure 2).

**TABLE 6:** Mean inhibition (percentage relative to the negative control) of the various concentrations of the egg yolk from different diets against PC3 cancer cell (*n* = 3, *p* < 0.05)

Concentration (μg/ml)					
Treatment	0 (negative control)	625	1250	2500	5000
Control (basal diet)	0^b^	1.80 ± 0.90^b^	6.22 ± 1.75^a^	6.97 ± 2.74^a^	6.72 ± 2.31^a^
Basal diet + 40 ppm of saffron supplement	0^c^	2.00 ± 0.53^c^	14.66 ± 2.65^a^	10.03 ± 1.76^b^	14.81 ± 4.52^a^
Basal diet + 60 ppm of saffron supplement	0^c^	20.56 ± 1.59^a^	21.00 ± 1.11^a^	18.84 ± 3.39^a^	10.56 ± 2.82^b^
Basal diet + 80 ppm of saffron supplement	0^e^	17.91 ± 0.76^d^	29.30 ± 3.79^b^	23.17 ± 2.63^c^	42.46 ± 1.21^a^

^a, b^The means with various letters in each row are significantly different (*p* < 0.05).

Based on the results the inhibition percentages on PC3 cancer cells in different concentrations were not significant in T1 and T2 diets. In T3 diet, the inhibition percentage of 625 μg/ml was significant compared to the negative control, which was about 20%. Although no significant difference was reported between 625, 1250 and 2500 μg/ml in terms of inhibition percentage (*p* ≥ 0.05), 20% inhibition at 625 μg/ml was considered as important since it has a very low concentration. The results may indicate that PC3 cells are very sensitive to even low concentrations of egg yolk from this diet, where the percentage of inhibition of egg yolk does not seem to be dose dependent. Regarding the T3 diet, at a concentration of 5000 μg/ml egg yolk, the positive effects of natural compounds in egg yolk may have benefited from the cancer cell growth as observed earlier with MCF‐7, which can overcome the anticancer effects of feeding laying hens with saffron petal extract. In the T4 diet, significant inhibition was observed at a concentration of 5000 μg/ml, which is in line with the results obtained in MCF‐7 cells (Figure 2).

A majority of pharmacological effects is related to the presence of active components in saffron petal, most of which exhibit antioxidant activities. Saffron petal is consisted of different active ingredients such as anthocyanins, flavonoles (kaempferol), new monoterpenoids including crocusatin‐J and 4‐dihydroxybutyric acid. Among the different isolated compounds, crocusatin‐K, crocusatin‐L, and 4‐hydroxy‐3,5,5‐trimethylcyclohex‐ 2‐enone show antityrosinase activity while protocatechuic acid, kaempferol, and kaempferol 7‐*O*‐*â*‐D‐glucopyranoside scavenge R,R‐diphenyl‐*â*‐picrylhydrazyl (DPPH) radicals more than R‐tocopherol. Based on the literature, plant polyphenolic flavonoids are one of the major groups of compounds acting as primary antioxidant free‐radical terminator (Hassani et al., 2014). The antidepressant activity of kaempferol, as an active compound of saffron petal, was evaluated in mice and rats by using forced swimming test. Kaempferol was injected intraperitoneally in mice (100 and 200 mg/kg) and rat (500 mg/kg), and compared with fluoxetine as a positive control (20 mg/kg). Kaempferol reduced immobility time in mice similar to fluoxetine (Hosseinzadeh et al., 2007).

Recently, some researchers have highlighted many therapeutic roles for saffron and its components such as anticancer, antitumour and antineuropathic properties (Amin & Hosseinzadeh, 2012; Hosseinzadeh, 2014; Hosseinzadeh et al., 2005). Furthermore, saffron plays an essential role in cell proliferation inhibition, apoptosis induction, antioxidant and free radical inhibitory effects, gene conservation, lipid peroxidation prevention, and anti‐inflammatory processes. The results of the above‐mentioned mechanisms indicated the potential therapeutic properties of saffron for liver, gastric, colorectal, pancreatic and ileum cancers (Khorasany & Hosseinzadeh, 2016). Given that crocetin is known as an anticancer compound, it exhibits antioxidant, antiproliferative and apoptosis activities against cancer cells (Bathaie et al., 2013). Reducing the level of malondialdehyde and promoting the level of glutathione and antioxidant enzymes such as superoxide dismutase, catalase, and glutathione peroxidase are the main functions of crocin against oxidative stress (Sun et al., 2014). Furthermore, it, as another active constituent of saffron, represents anticancer effects similar to crocetin (Hoshyar et al., 2013). The saffron ethanolic extract can induce apoptosis in tumour cells. Tavakkol‐Afshari et al. (2008) proved the specific role of saffron in the death of human liver (HepG2) and cervical (HeLa) cancer cells. The researchers have outlined two mechanisms leading to the anticancer properties of saffron. The mechanisms involve inhibiting cell proliferation in the early stages of cell growth by inducing cell cycle arrest or probably targeting DNA sequences and modulating gene expression, as well as removing cancer cells through apoptosis (Amin et al., 2011).

Saffron has a selective toxicity against cancer cells, with mechanisms including inhibition of RNA and DNA synthesis and increasing apoptosis. Crocin is considered as the most important anticancer agent of saffron, the effect of which may be related to the changes in gene expression and apoptosis induction in cancer cells. Crocetin has an inhibitory effect on the growth of cancer cells, the effect of which may be related to the reduced synthesis of DNA, RNA and protein in neoplastic cells, inhibition of RNA polymerase II, and interaction with histone H1 and H1‐DNA structures. In addition, saffron and its crocin, along with crocetin have demonstrated anticancer and cancer‐preventive effects in animal models of cancer. Furthermore, saffron and its components do not affect normal cells (Milajerdi, et al., 2015). However, conducting further research is necessary for testing these effects on several normal human cell lines.

Recently, Amin et al. (2021) studied anticancer effects of safranal in hepatocellular carcinoma (HCC) and indicated a DNA damage repair and apoptosis unique safranal‐mediated cell cycle arrest at G2/M phase and a pronounced effect on DNA damage machinery. Further, safranal activated both intrinsic and extrinsic initiator caspases where ER‐stress was evidently a major mediator. The gene set enrichment analysis revealed that unfolded protein response (UPR) is among the top up‐regulated genes in the presence of safranal. Finally, crocin can prevent adverse cisplatin and cyclophosphamide effects such as oxidative damage, inflammation, and organ toxicity (e.g. hepatotoxicity).

## 4 ANTIOXIDANT STATUS

The antioxidant effects of saffron petal extract in laying hens are reported in Table 7. According to the results of this experiment, malondialdehyde, DPPH and SOD levels were significantly affected by experimental treatments (*p* <0.05). Malondialdehyde was significantly decreased in treatments containing saffron at 60 and 80 ppm compared to the control and saffron at 40 ppm (*p* < 0.05).

**TABLE 7:** Effects of saffron petal hydroalcoholic extract on Antioxidant activity

Treatment	SOD (U/ml)	MDA(%)	DPPH(%)
Control (basal diet)	201.70^b^	96.56^a^	76.33^b^
Basal diet + 40 ppm of saffron supplement	216.80^b^	93.80^a^	82.50^a^
Basal diet + 60 ppm of saffron supplement	341.90^a^	87.63^b^	87.50^a^
Basal diet + 80 ppm of saffron supplement	363.50^a^	85.30^b^	88.33^a^
SEM	0.776	6.12	7.24
*p* Value	0.0001	0.0001	0.0001

SEM: standard error of measurement.

^a,b^Values in a row with no common superscript letter are significantly different (*p* < 0.05).

DPPH, diphenyl‐1‐picrylhydrazyl.

Also, the percentage of DPPH in experimental treatments, especially diets containing saffron, increase significantly compared to the basic diet (*p* < 0.05). Also, the amount of SOD in the control diet compared to other diets and the control showed a significant decrease (*p* < 0.05). SOD, the primary antioxidant enzyme that protects cells from oxidative stress, increases the antioxidant ability to eliminate overactive reactive oxygen species (ROS) and reduce lipid peroxidation in poultry (Zhu et al., 2017). In addition, it increased serum antioxidant status by increasing T‐AOC content, T‐SOD and Mn‐SOD activities and decreasing MDA content (Zhang et al., 2020). Eggs can be enriched with antioxidants through manipulating poultry feed (Surai et al., 2006).

## 5 CONCLUSION

In general, the addition of saffron petal hydroalcoholic extract into diet leads to an improvement in egg production percentage (at 80 ppm in layers diet) and egg quality (yolk colour and Haugh unit). Further, a significant effect of the diet on the growth/inhibition of MCF7 and PC3 cancer cells was observed in a positive concentration‐dependent manner (*p* < 0.05). Some treatment levels indicated a dose dependent effect on the cancer cell inhibition, with higher inhibition observed at lower concentrations of egg yolk and some reduced effects at higher concentrations. Like the results of the previous studies, some egg yolk components actually could support the growth of cancer cells when used at higher concentration levels. Therefore, it is recommended to consume the diet of saffron petal at 80 ppm in layers. Although this level of inhibition is below 50% and egg yolk cannot naturally be considered as an anticancer drug, egg yolk is a human food (not a drug). Thus, its food safety requires that the bioactive effects should not have too much bioactivity. Finally, malondialdehyde, DPPH and SOD significantly improved by treatments including saffron petal hydroalcoholic extract . On the basis of these observations, we conclude that saffron petal hydroalcoholic extract can be used as a new feed additive to potentially modulate the antioxidant status of laying hens and improve their production performance and egg quality and the inhibitory effect on cancer cells, best achieved with 60–80 ppm extract.

## AUTHOR CONTRIBUTIONS

Study concept and design: R.V. Acquisition of data: M.T. Analysis and interpretation of data: M.E.T. Drafting of the manuscript: R.V. Critical revision of the manuscript for important intellectual content: R.V. Statistical analysis: M.E.T. Administrative, technical and material support: Study supervision: R.V. and M.T. Study supervision: R.V.

## CONFLICT OF INTEREST

The authors declare that there is no conflict of interest.

## DECLARATIONS

The authors are faculty member at Islamic Azad University listed on the manuscript are employed by a non‐government agency that has a primary function research and/or education. The authors are not submitting this manuscript as an official representative or on behalf of the government.

## ETHICAL STATEMENT

We hereby declare all ethical standards have been respected in preparation of the submitted article.

### PEER REVIEW

The peer review history for this article is available at https://publons.com/publon/10.1002/vms3.910

## Data Availability

The data that support the findings of this study are included in this published article.
